# Exploring Gynecological Pathology and Imaging: A Crossroads of Technology, Biology, and Care

**DOI:** 10.3390/diagnostics15151976

**Published:** 2025-08-07

**Authors:** Graziella Di Grezia

**Affiliations:** Department of Life Sciences, Health and Healthcare Professions, Link Campus University, 00042 Rome, Italy; g.digrezia@unilink.it

The field of gynecologic diagnostics is undergoing a profound transformation. No longer confined to traditional imaging and histopathology, it is now being reimagined through the integration of advanced technologies, interdisciplinary collaboration, and an increasingly patient-centered clinical lens.

This Special Issue of *Diagnostics*, “Exploring Gynecological Pathology and Imaging,” gathers nine contributions that reflect this evolution. The published articles span breast imaging physics [contributions 1, 2], cervical cancer prevention strategies [contribution 3], fibroid diagnosis pathways [contribution 4], applications of artificial intelligence in urogynecology [contribution 5], standardized approaches to vulvar ultrasound [contribution 6], already cervical cancer prevention strategies [contributions 7, 8], and even the prognostic potential of PET/CT parameters in ovarian cancer [contribution 9]. Together, these works show that innovation in gynecological imaging and pathology is no longer optional—it is foundational.

Some contributions offer fresh clinical perspectives on long-debated challenges. For example, the study by Cantatore et al. [contribution 8] proposes a novel risk stratification model for recurrence in patients treated for high-grade cervical intraepithelial neoplasia. This model integrates HPV persistence, margin status, and inflammatory markers—moving decisively beyond single-parameter evaluations. Similarly, Tsampazis et al. [contribution 3] assess the diagnostic accuracy of HPV mRNA testing and immunohistochemical markers (p16/Ki67), suggesting meaningful refinements in colposcopic triage.

Technological and methodological innovation are also evident in our own contributions to this issue [contributions 1, 2], in which we explored the foundational physics of mammography, tomosynthesis, and contrast-enhanced imaging. Our aim was to make complex technical knowledge clinically accessible, thereby supporting more nuanced decision-making in breast diagnostics.

Beyond image acquisition and interpretation, this Special Issue highlights how AI [contribution 5], self-sampling technologies [contribution 7], and hybrid diagnostic algorithms can amplify access and precision. In doing so, it echoes the growing global consensus that gynecologic care must become more inclusive, predictive, and adaptive.

From a personal perspective, the themes of this Special Issue are closely aligned with my own research over the past decade, which has addressed the clinical relevance of radiologic tools, cost-effective imaging strategies, and psychological–phenotypic correlates in breast disease. I am particularly interested in how hybrid human–machine systems—grounded in radiomics and intelligent imaging—can transform diagnosis into a dynamic process of care rather than a static endpoint. Radiomics, in particular, bridges the gap between medical images and personalized medicine by extracting quantitative data that inform clinical decisions [1,2].

This collection of articles is not the end of a journey, but a new point of departure. My deepest thanks go to all the contributing authors, the reviewers for their generous insight, and the editorial staff at *Diagnostics* for their professional support. Moreover, addressing potential biases in artificial intelligence systems is crucial to ensure equitable and effective healthcare delivery [3].

We hope this Special Issue will serve as an open window on the future of women’s health diagnostics: a space where biology, data, and clinical wisdom converge. The advances in deep learning and hybrid imaging approaches promise to enhance screening accuracy and clinical outcomes, especially in breast cancer detection and management [4,5].
